# Saltwater-responsive bubble artificial muscles using superabsorbent polymers

**DOI:** 10.3389/frobt.2022.960372

**Published:** 2022-08-29

**Authors:** Daniel Gosden, Richard Suphapol Diteesawat, Matthew Studley, Jonathan Rossiter

**Affiliations:** ^1^ Department of Engineering Mathematics, University of Bristol, Bristol, United Kingdom; ^2^ Department of Engineering Design and Mathematics, University of the West of England, Bristol, United Kingdom

**Keywords:** artificial muscle, superabsorbent polymer, hydrogel, sodium polyacrylate, soft robotics

## Abstract

Robots operating in changing underwater environments may be required to adapt to these varying conditions. In tidal estuaries, for example, where the degree of salinity cycles in step with the level of the water, a robot may need to adapt its behaviour depending on the position of the tide. In freshwater bodies, the unexpected presence of a pollutant may also require the robot to respond by altering its behaviour. Embodying this sensing and response in the body of the robot means that adaptivity to the environment can be achieved without resorting to centralised control. This can also allow direct responsivity using ‘free’ environmental energy, actuating without requiring stored onboard energy. In this work we present a soft artificial muscle, the contraction of which varies in response to the salinity the water surrounding it. The novel actuator uses a super-absorbent polymer gel encapsulated within a series of discrete cells. This gel readily absorbs water through the membrane wall of the actuator, and can swell to over 300 times its initial volume. This swelling generates significant pressure, changing the shape of the cells and driving the contraction of the muscle. The degree of swelling is significantly reduced by the presence of salts and pollutants in the surrounding water, so transitioning from a freshwater to a saltwater environment causes the muscle to relax. In this paper, we discuss the design and fabrication of these superabsorbent polymer-based Bubble Artificial Muscle (SAP-BAM) actuators. The tensile properties of the muscle under actuated (fresh water) and relaxed (salt water) conditions are characterised, showing a maximum generated force of 10.96N. The length response under constant load for a full actuation cycle is given, showing a maximum contraction of 27.5% of the initial length at 1N load, and the performance over repeated actuation and relaxation cycles is shown. The SAP-BAM muscles are straightforward to fabricate and are composed of low-cost, freely-available materials. Many existing pneumatically-actuated muscles can be modified to use the approach taken for this muscle. The muscle presented in this work represents the first example of a new class of super-absorbent polymer-driven environmental soft artificial muscles.

## 1 Introduction

Underwater environments can be highly changeable, and a robot operating in such an environment will need to be able to adapt to robustly handle these changes. For example, a robot situated in a tidal estuary may have the purpose of controlling the release of some agent into the water (for example to control farm nitrate runoff) over an extended period of time. Such a robot would need to adapt to differences in the water level, the speed and direction of flow, and the salinity of the water. Driven by the tide, these conditions change over the course of hours. Since the robot may be operating in such an environment for a long duration, it becomes attractive to use energy drawn from its surroundings for such adaptations, rather than using stored onboard energy. Moreover, if we can embody such adaptations in the body of the robot then such adaptations can occur in direct response to the environmental changes, rather than signalled through some centralised control. When the environmental change is slow, the response need not be rapid, so long as it occurs on a faster timescale than the changes in the environment.

Hydrogels lend themselves naturally to direct stimuli-responsive actuation, particularly in aqueous environments. These materials are typically soft and capable of holding many times their own mass in water (Oyen, 2014). Many hydrogels are stimulus-responsive (for example, against heat (Feng et al., 2019), light (Jiang et al., 2020), or pH (Kocak et al., 2017)), and consequently are a topic of ongoing investigation for use in soft artificial muscles (Park and Kim, 2020) and robotics (Lee et al., 2020). There are limitations to the use of these materials, however: the high degree of softness often makes them weak, and though very fast actuation in the forward direction has been demonstrated Li et al. (2020), they are often slow to recover. Moreover, actuators based purely on hydrogels without additional structure generate forces that do not scale well to larger actuators (Na et al., 2022).

To mitigate some of these drawbacks, hydrogel-based artificial muscles have previously been investigated. Similar to the use of pressurised air or fluid in pneumatic or hydraulic muscles (Tiwari et al., 2012), these hydrogel-based muscles use the swelling of the gels to drive the contraction of the muscle. A McKibben-type actuator filled with different hydrogels was investigated by Santulli et al. (2005), although the reversibility of their actuators was not demonstrated. Salahuddin et al. (2020) show thermally-reversible actuation in a similar braided actuator filled with hydrogel beads.

For actuators of this type, the speed and extent of contraction are dependent on the degree to which the encapsulated hydrogel can swell. Superabsorbent polymers (SAPs) are a class of hydrogel which exhibit an extremely high degree of swelling (Ma and Wen, 2020). One commonly-used SAP, sodium polyacrylate, can swell to more than 600 times its initial volume (Liu and Guo, 2001), making this a promising material for use in encapsulated hydrogel-based artificial muscles. Motivated by this high swelling factor, Velders et al. (2017) and Jain et al. (2021) have investigated the use of glued commercially-available sodium polyacrylate beads in bending and contractile actuators, but do not look into the reversibility of this approach.

**FIGURE 1:**
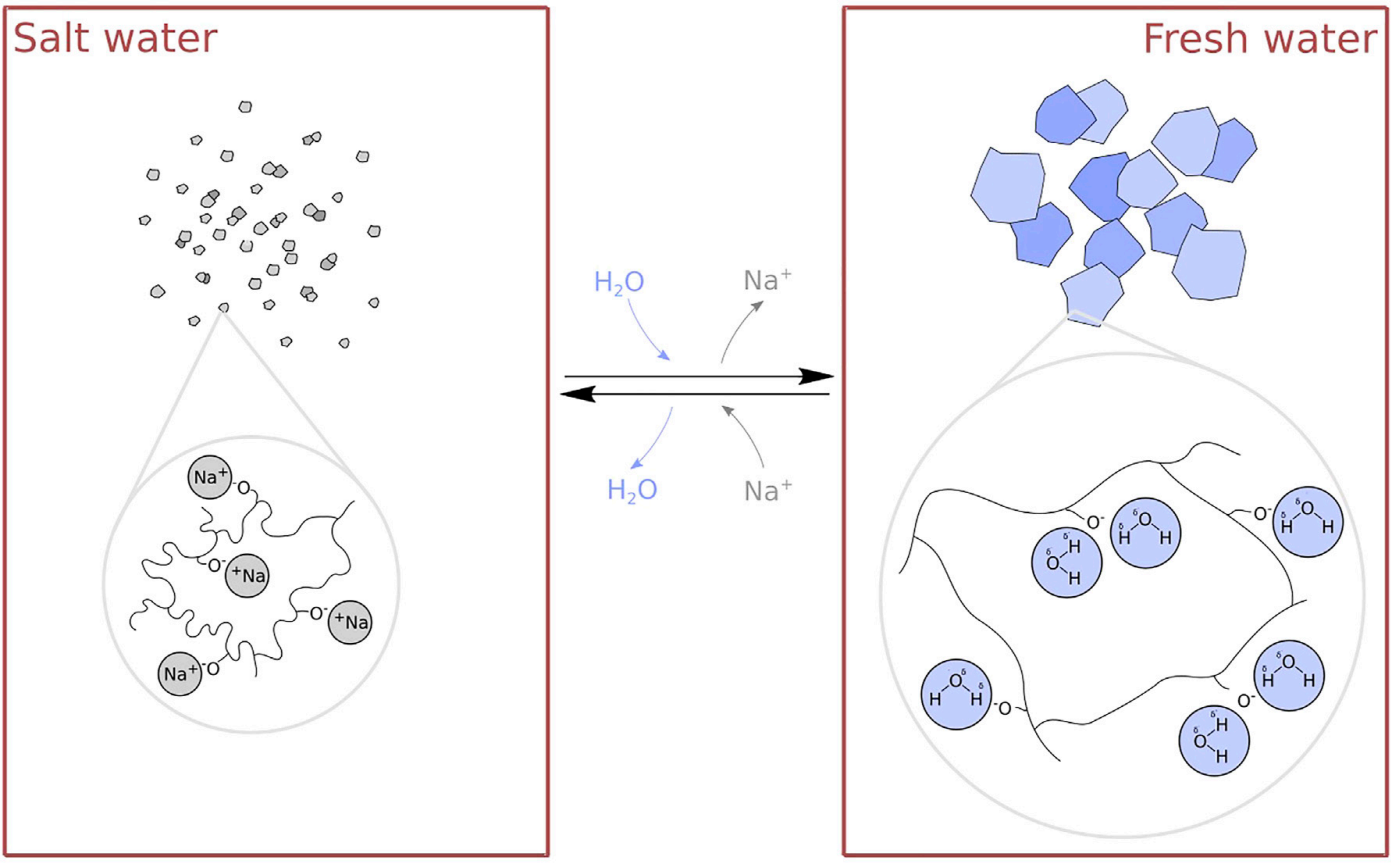
The structure and swelling behaviour of sodium polyacrylate. In salt water, sodium ions (Na^+^) interfere with water (H_2_O) uptake and significantly reduce the swelling of the gel (left). In salt-free environments, sodium ions diffuse out of the gel, which is then capable of holding a large amount of water (right).

In the presence of pollutants or salts - for instance in salt water - the degree of swelling of sodium polyacrylate is significantly reduced (Ma et al., 2004). Gel that has been swollen in fresh water will therefore shrink when placed into salt water. A high degree of swelling is recovered when the gel is returned to fresh water (see Figure 1). This raises the opportunity for the design of novel saltwater-sensitive actuators using these gels.

In this paper, we present an environmentally-reactive encapsulated SAP-based soft artificial muscle. Since the degree of contraction varies in response to the salinity of the water surrounding it, the muscle is well-suited for use in variable-salinity environments. In the following section we discuss the design and fabrication of this actuator. The tensile properties of the actuator in both actuated and relaxed states are then determined, and the length response during an actuation-relaxation cycle is characterised. We also demonstrate the performance over repeated actuation cycles. Finally we give a comparison of our actuator’s performance against other encapsulated-hydrogel actuators, and discuss its applicability for use in a marine soft robot.

## 2 Actuator design

### 2.1 General design principles

**FIGURE 2:**
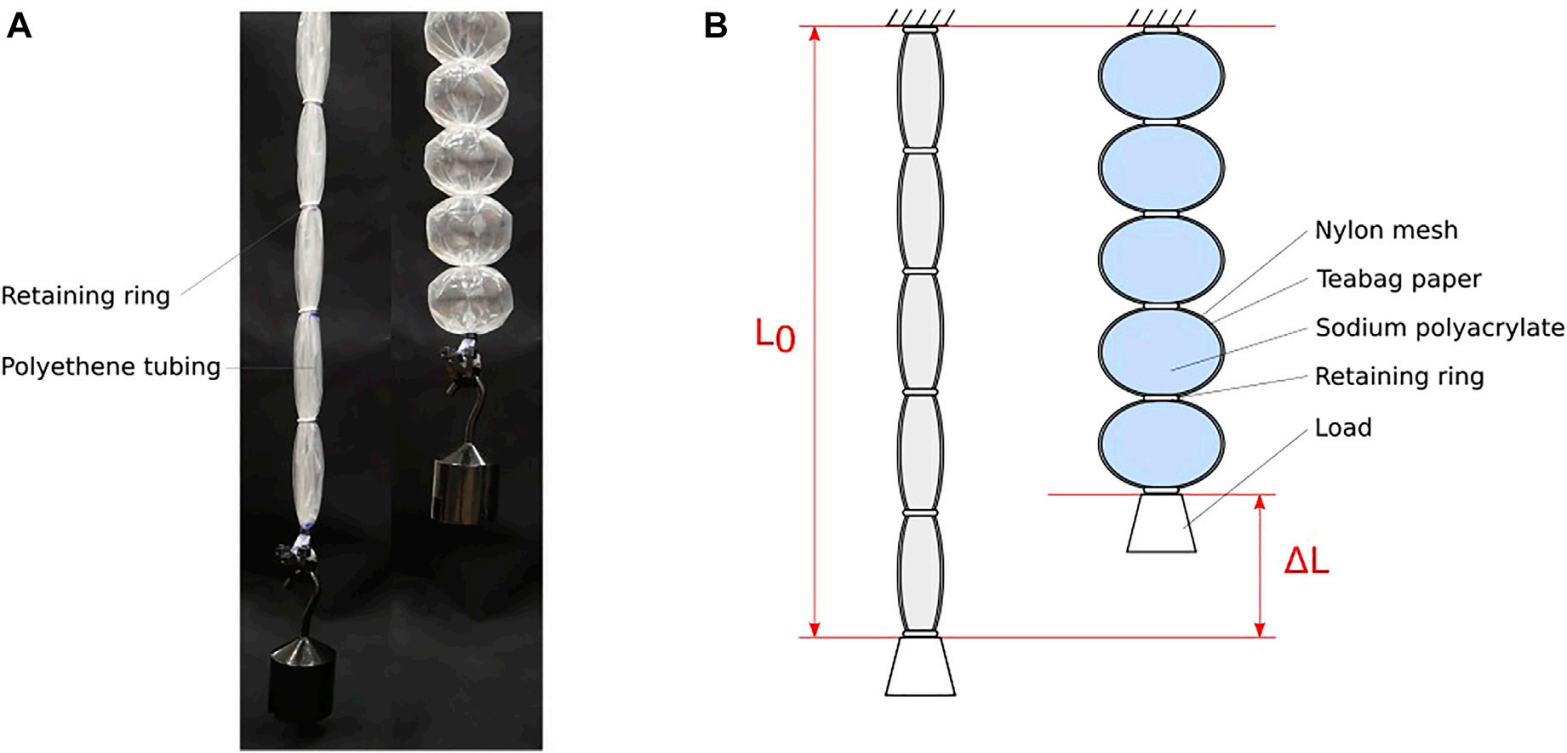
**(A)** Pneumatic BAM actuator relaxed (left) and contracted (right). Images reproduced with permission from Diteesawat et al. (2021) **(B)** SAP-BAM design. Left shows the actuator in salt water (relaxed), right shows the actuator in fresh water (contracted).

The actuator presented in this work takes inspiration from the pneumatic Bubble Artificial Muscle (BAM) presented by Diteesawat et al. (2021). In those actuators, flexible airtight tubing is constrained in diameter at intervals along its length, producing a series of inflatable cells (see Figure 2A). When the inside of the tube is pressurised, these cells bulge and shorten, causing an overall contraction in the muscle.

For the new actuators presented in this work, the contraction is instead driven by the swelling of a super-absorbent polymer, sodium polyacrylate, within each of the cells. The tubing is constructed by a permeable material which allows liquid to pass into and out of the cell whilst preventing escape of the gel. The design of the new superabsorbent polymer-based Bubble Artificial Muscle (SAP-BAM) can be seen in Figure 2B.

To ensure maximum contraction of a BAM-type muscle, the spacing between the retaining rings must be optimised, as shown by Daerden (1999). The optimal spacing between the rings *L*_*optimal*_ is dependent on the chosen tubing radius *R*_*material*_ and ring radius *R*_*ring*_:

$$L_{\mathrm{optimal}}=\frac{R_{\mathrm{ring}}}{\sqrt{0.5}\cos\phi_{R,L_{\mathrm{optimal}}}}F\left(\phi_{R,L_{\mathrm{optimal}}}\backslash 0.5\right)$$ (1)

where *F* provides the elliptic integral of the first kind, and $\phi_{R,Loptimal}=\arccos\frac{R_{\mathrm{ring}}}{R_{\mathrm{material}}}$ .

For the actuators used in this work, a tubing radius of 9.5 mm and ring radius of 2 mm were used, providing an optimal ring spacing of approximately 21 mm. With these parameters, the volume of each bubble at maximum contraction, *V*_max_ is 2.35 cm^3^.

### 2.2 Sodium polyacrylate swelling

An important variable in the construction of the SAP-BAM actuators is the amount of SAP used in each ‘bubble’ cell. Too little polymer will not provide the full contractile range possible - too much and the actuator may rupture. To determine an appropriate mass of polymer to use, it is important to know by how much the polymer is liable to expand after a given amount of time in solution. The extent and speed of swelling of superabsorbent polymers is dependent on a number of factors: the degree of crosslinking, homogeneity of the polymer, and granularity of its powdered form all play a part. Nevertheless, the swelling rate and maximum volume are typically represented well by an exponential decay model (Omidian et al., 1998):

$$SF_{m,t}=\frac{m_{t}}{m_{0}}=SF_{m,\infty }\left(1-e^{-t/\tau }\right)$$ (2)

where *SF*_*m*,*t*_ is the mass swelling factor at time *t*; the ratio between the mass at time *t* (*m*_*t*_) and the initial mass (*m*_0_). The ultimate swell factor *SF*_*∞*_ and the time constant *τ* can be determined empirically.

**FIGURE 3:**
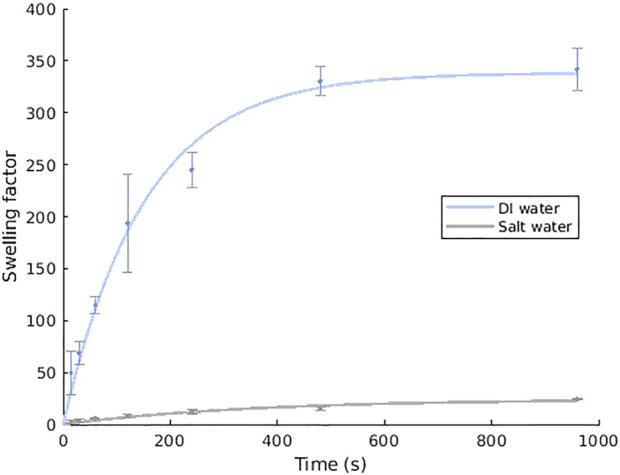
The swelling behaviour of sodium polyacrylate powder in deionised and salt water (4% w/w). The fitted curves use *SF*_*m*,*∞*_ = 338.26 and 24.37, and *τ* = 151.06 s and 352.95 s for deionised water and salt water respectively. Error bars show one standard deviation.

An established method to gather swelling data is using vacuum filtration. To characterise the polymer used in the current work, the procedure outlined by Zhang et al. (2020) was followed. For each test, 25 mg dry polymer was placed for the desired amount of time into 50 ml of either deionised (DI) water or 4% NaCl solution. Each measurement was repeated three times. The results for the sodium polyacrylate super-absorbent polymer used in this paper can be seen in Figure 3, which was used to calculate *SF*_*∞*_ and *τ* using a curve fitting method, and thus calculate *SF*_*m*,*t*_ at any *t*.

The volume swelling factor *SF*_*v*_ of the gel relates directly to the mass swelling factor, and can be given by:

$$SF_{v,t}=\frac{V_{t}}{V_{0}}=SF_{m,t}\frac{\rho_{p}}{\rho_{l}}+1$$ (3)

where *V*_*t*_ is the volume at time t, and *ρ*_*p*_ and *ρ*_*l*_ are the density of the dry polymer and the liquid it is placed in, respectively. With *ρ*_*p*_ = 0.54 g/cm^3^, *ρ*_*l*_ = 1.00 g/cm^3^, and *SF*_*m*,*∞*_ = 338.26, we can compute the initial mass of polymer in each bubble required to produce a volume of 2.35 cm^3^ (maximum contraction) as *m*_0_ = 6.91 mg. However, with this quantity of gel, swelling to the maximum volume would take a long time–given the exponential swelling rate described above–and this value does not account for the compression the gel would undergo within the actuator. To compensate for this, 25 mg of polymer is instead used per bubble in our actuator. This is sufficient to generate faster contraction without rupturing the wall of the actuator.

### 2.3 Fabrication

**FIGURE 4:**
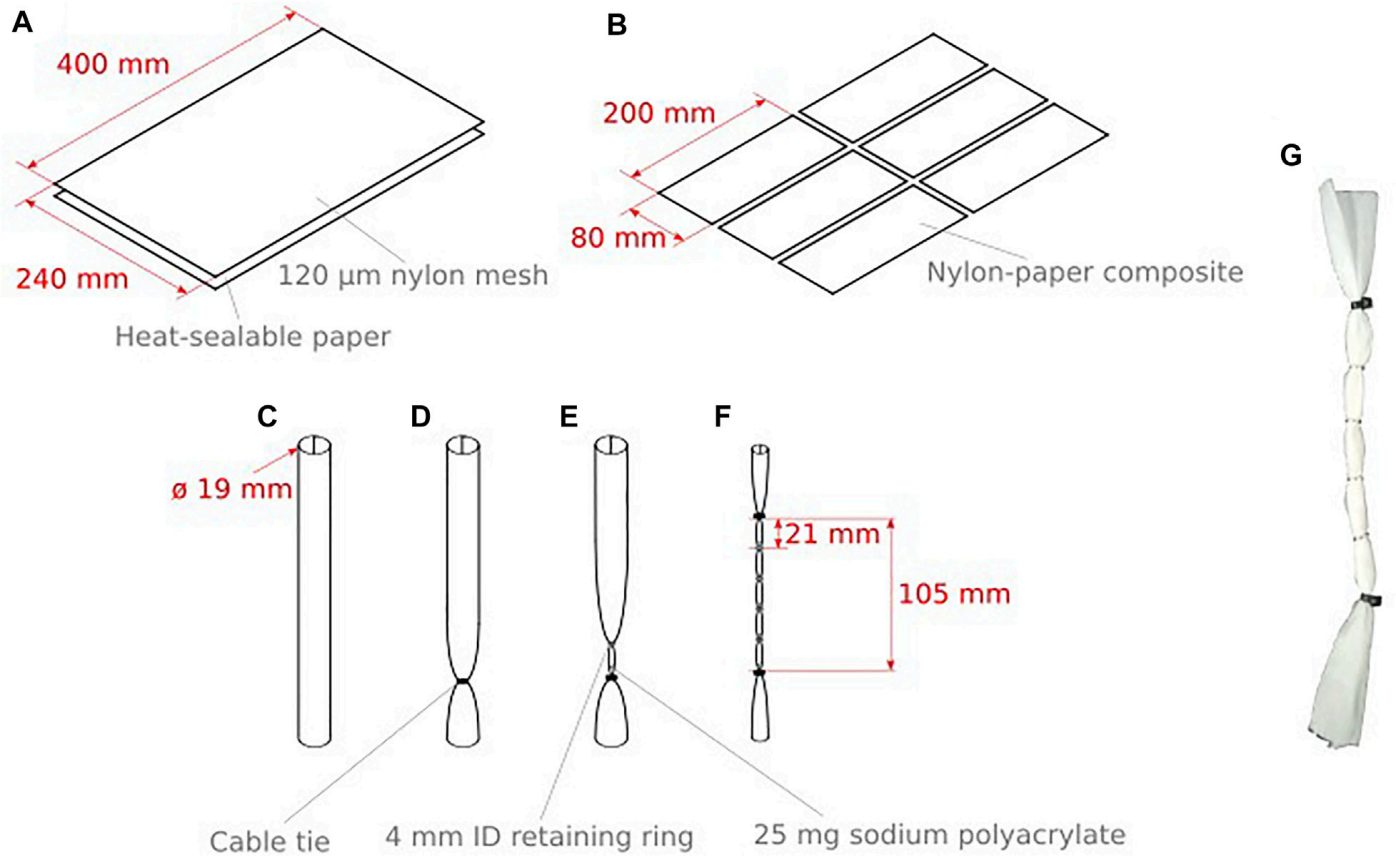
Fabrication steps for the SAP-BAM actuator. **(A,B)** Nylon mesh and ‘teabag’ fabric are heat sealed together and cut to size **(C–F)** The sheets are sealed into a cylinder and ‘bubbles’ filled with SAP powder are made, separated by retaining rings. **(G)** An assembled SAP-BAM actuator.

The fabrication process for the SAP-BAM artificial muscles can be seen in Figure 4. The main material for construction of the actuator is a sheet material composed of a layer of nylon mesh (Nitex nylon mesh 120 μm, Scientific Laboratory Supplies) and a layer of heat-sealable paper (‘Tea bag paper’, Art Van Go). The nylon mesh and paper are laminated together using a CNC heat-sealer. An equilateral grid pattern of sealing points was used, with 16 mm between each point (seeSupplementary Material S1).

Each five-bubble SAP-BAM used for this work was assembled from an 80 mm × 200 mm sheet of this material, four 4 mm diameter steel retaining rings, 125 mg total sodium polyacrylate powder (432,,784, Sigma Aldrich) divided equally between the five bubbles, and two cable ties (RS Pro 100 mm × 2.5 mm, RS).

The nylon-paper material was heat-sealed along the long edge to form a 9.5 mm diameter cylinder. This was arranged so that the nylon side of the sheet was on the outside of the cylinder. The cylinder was then firmly sealed 4 cm from the bottom end using a cable tie. To form the first bubble, 25 mg sodium polyacrylate powder was carefully placed into the long end of the cylinder and a retaining ring positioned 21 mm from the tie. This ring is held in place by friction from the nylon-paper material. The process of adding sodium polyacrylate powder followed by the placement of a retaining ring at a spacing of 21 mm from the previous ring was repeated for the remaining rings and powder, with the last bubble being closed with a second cable tie. An assembled actuator can be seen in Figure 4G.

## 3 Characterisation

### 3.1 Materials and methods

**FIGURE 5:**
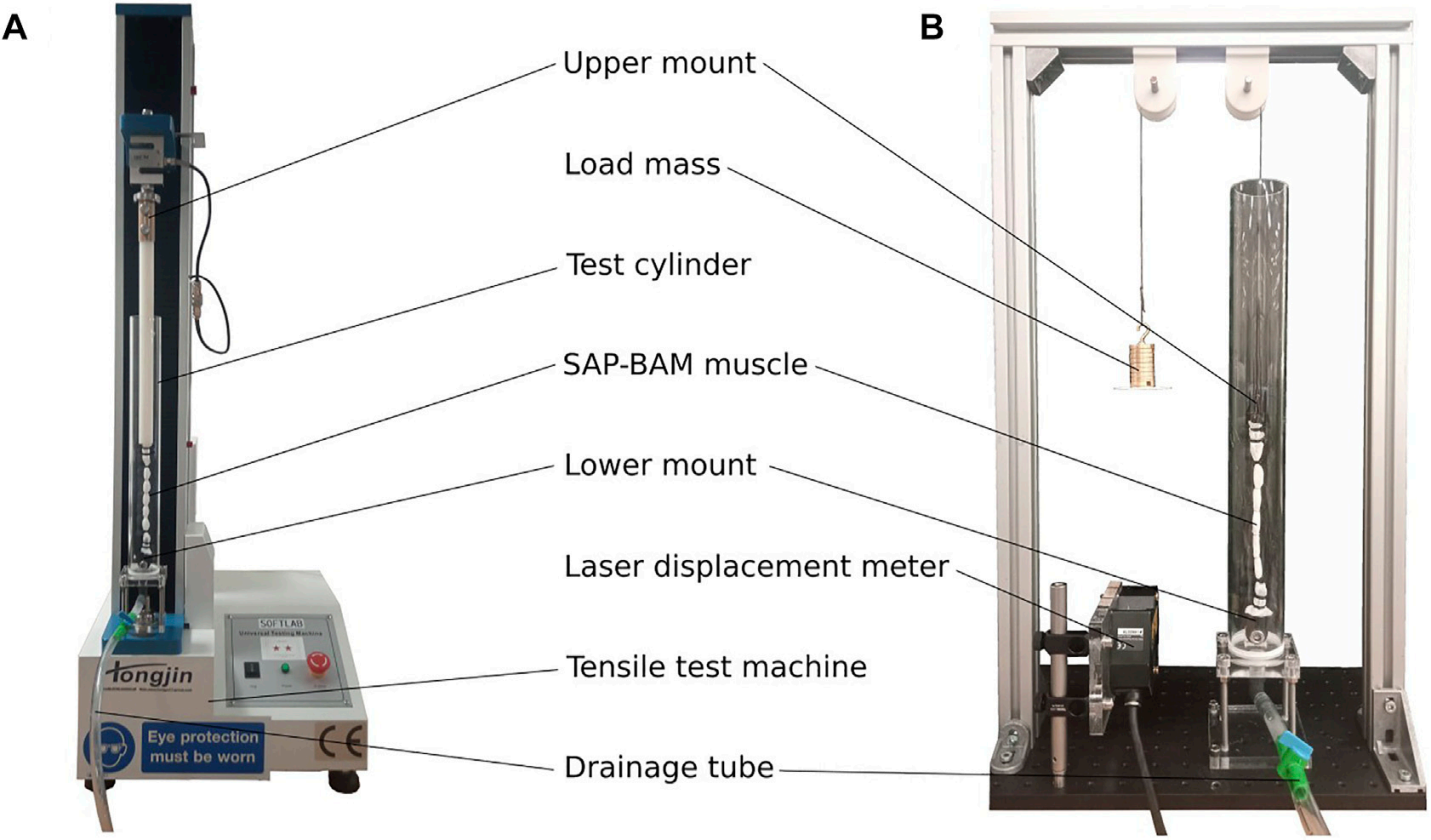
Experimental setup for SAP-BAM characterisation. **(A)** Force-extension testing setup **(B)** Isotonic testing setup.

Tests were performed to characterise the tensile properties of the SAP-BAM and its response under isotonic conditions. A similar experimental setup was used for each of the tests (see Figure 5). A new actuator was prepared as described in Section 2.3 for each test.

At any given time for each of the tests performed, the actuator was submerged in a quantity of either fresh water (which would contract the actuator), or salt water (which would cause the actuator to relax). For the fresh water solution, deionised water (CHE3876, Scientific Laboratory Supplies) was used. For the salt water solution, a 4% w/w solution of sodium chloride (S/3120/60, Fisher Chemical) in deionised water was used to provide an approximation of the salinity range seen in the natural estuarial environment.

Due to the limited size of the test cylinder, in all tests when transitioning from a salt (relaxed) state to a fresh (contracted) state, it was necessary to perform a washing routine to remove excess salt. For this, the (initially salty) water was removed and replaced with fresh water, and then left for a 5 minute interval. This washing was repeated two further times.

In every test, the cylinder was filled to the same level. In the isotonic tests this required 500 ml water. For the force-extension tests a smaller volume of 400 ml was required.

#### 3.1.1 Force-displacement

Force-displacement tests to determine the mechanical properties of the actuator were conducted with a universal testing machine (Hongjin Test Instrument Co.) with a 100N load cell. The experimental setup can be seen in Figure 5A. The crosshead speed was 10 mm/min, and each sample was tested until either failure or until the force limit (75N) was reached.

For tests on the contracted actuator, a fresh actuator was placed into an excess of deionised water prior to testing and left for at least 16 h to equilibriate. After this time, the length of the actuator was recorded and the actuator fixed into the test apparatus. The cylinder was filled with deionised water and allowed to rest for a further 5 min before commencing the test.

For tests on the relaxed actuator, a similar procedure was followed with salt water used in place of fresh water.

For both the contracted and relaxed actuator the procedure was repeated four times with a fresh actuator used for each test, and the mean force reading was calculated across these samples.

#### 3.1.2 Isotonic actuation and relaxation

Three test regimes were used: one to characterise actuation, the second to show relaxation, and the final test across a full actuation-relaxation cycle. The same test apparatus was used for each of these, as shown in Figure 5B. For this test, loads of 1 N and 1.96 N were chosen. These are approximately 10% and 20% respectively of the maximum force generated in the force-displacement tests (see Section 3.2.1). The loads were applied to the actuator as suspended masses on a pulley system. The displacement of the actuator was determined by measuring the vertical position of the mass with a laser displacement meter (LK-G152, Keyence Corporation).

In the actuation test, the actuator was initially equilibriated in salt water for at least 16 h. The salt water was then replaced with fresh water (after washing), causing the actuator to contract.

In the relaxation test, the actuator was initially equilibriated in fresh water for at least 16 h. The fresh water was then replaced with salt water, causing the actuator to relax.

In the full-cycle test, the actuator was initially equilibriated in salt water for at least 16 h. The salt water was then replaced with fresh water (after washing), causing the actuator to contract. After 250 min, the fresh water was again replaced with salt water, causing the actuator to relax.

Each of these tests was repeated three times, with a fresh actuator used on each of these tests, and the mean displacement was calculated across these samples.

#### 3.1.3 Repeated cycles

Testing to show behaviour under repeated contraction-relaxation cycles also used the isotonic setup shown in Figure 5B.

For this test, the actuator was initially equilibriated in salt water (the relaxed state) for at least 16 h. After this time, the salt water was then replaced (after washing) with fresh water to cause the actuator to contract. The actuator was left in this fresh water for 2000 s (including the time required for washing), after which the fresh water was replaced with salt water and left for a further 2000 s before being again replaced with fresh water. This fresh-salt-fresh cycling was repeated for six full cycles.

### 3.2 Results

#### 3.2.1 Force-displacement

**FIGURE 6:**
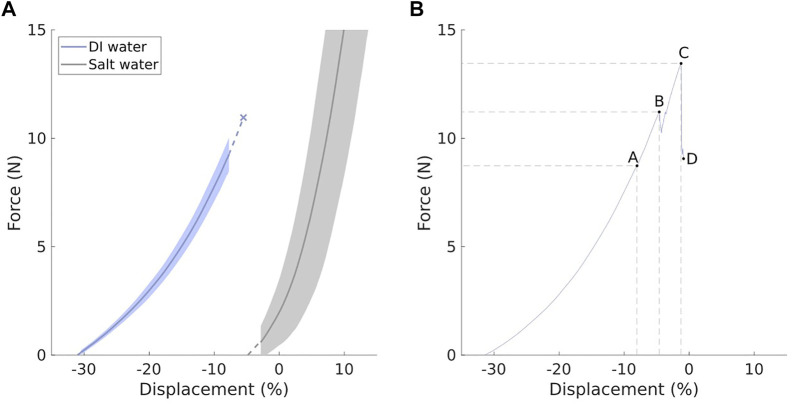
Results of SAP-BAM tensile testing. **(A)** Mean results (solid lines) for testing of SAP-BAM in fresh (DI) water (left) and salt water (right) conditions. Error bands show one standard deviation in the force reading. **(B)** Representative curve for one test in fresh water conditions. Labelled points show regions of interest, as discussed in section 3.2. Displacement in both **(A)** and **(B)** is calculated as a percentage relative to the length of newly-fabricated (dry) actuators.

Results from the force-extension testing can be seen in Figure 6. The displacement is calculated relative to the length of the newly-fabricated (dry) actuator. The displacement becomes negative when the actuator contracts.

Using graphical methods as demonstrated by Spinks et al. (2019), we can determine actuation parameters of the SAP-BAM. For the contracted actuator (actuated in DI water), the free stroke of the actuator is 26.1%. The mean yield force of the actuator in its contracted state was 10.96 N. The blocked force generated cannot be calculated accurately because the actuator would break at these forces.

The point at which an actuator was considered to have failed was not defined by complete mechanical failure (as is typical for materials yield testing), but rather by irreversible damage as a consequence of the gel escaping from the actuator wall. To highlight this, Figure 6B shows a single representative force-displacement curve which indicates various points of failure. Point A shows a small perturbation due to slippage of a retaining ring during tension. Point B shows a drop in force as the actuator wall partially ruptures and gel escapes. This escape of gel is irreversible, and so this is considered to be the yield point of the actuator. However, the general trend of the curve continues to point C, at which the tear in the actuator wall is large enough for the actuator to lose all strength. The force then drops off until point D, when the test was terminated.

#### 3.2.2 Isotonic actuation and relaxation

**FIGURE 7:**
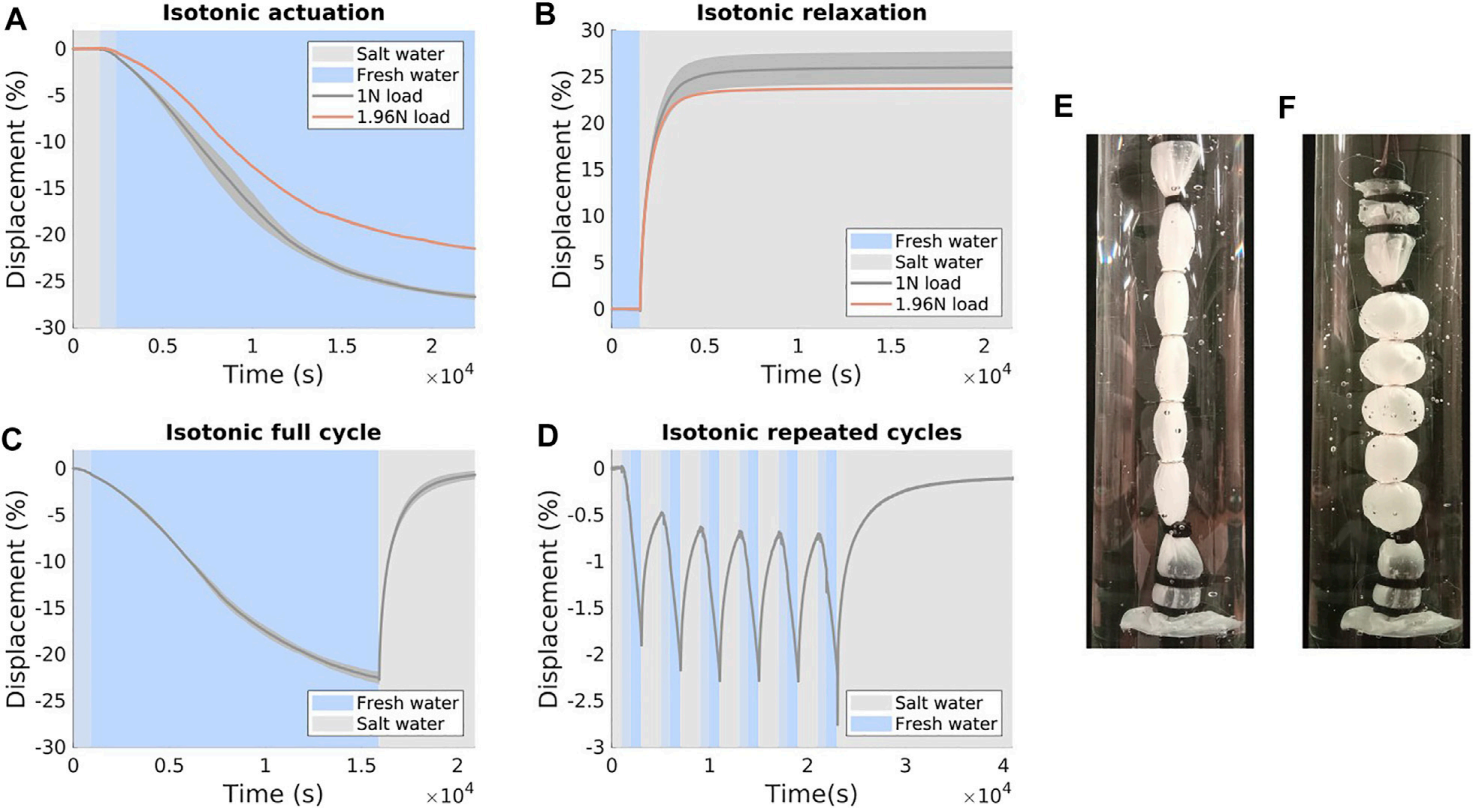
Results of isotonic testing. Where applicable, solid lines present mean values and error bands show one standard deviation in force, and displacement is calculated relative to the length of the dry actuator, zeroed at the start of the recorded data. **(A)** Actuation (salt-fresh transition) **(B)** Relaxation (fresh-salt transition) **(C)** Full cycle (salt-fresh-salt transition) under 1N load **(D)** Repeated cycling under 1N load **(E)** A relaxed actuator (in salt water) under 1N load **(F)** A contracted actuator (in fresh water) under 1N load.

Results from the isotonic actuation and relaxation tests can be seen in Figures 7A–C. Displacement was calculated relative to the length of the newly-fabricated (dry) actuator, then zeroed according to the state at the beginning of each test.

An actuator left to contract for more than 24 h total time reached a final displacement of 27.5%, which we can consider to be close to full contraction. Using this value, we can see that the actuator typically reaches 95% of its final contraction in ∼5 h.

The relaxation stroke is significantly faster and the actuator typically reaches 95% of its final displacement in ∼45 min.

#### 3.2.3 Repeated cycles

Results from the repeated cycles under isotonic conditions can be seen in Figure 7D. In these tests the cycling period was of sufficiently short duration to stop the artificial muscle reaching full contraction or relaxation in any cycle.

In this test the actuator reached a stable stroke pattern within three cycles. After these cycles were complete, the actuator recovered close to its initial displacement.

## 4 Discussion

A comparison of the proposed SAP-BAM environmentally-reactive artificial muscle with the pneumatic BAM and known encapsulated-hydrogel actuators from the literature can be seen in Table 1. It can be seen that the maximum contraction exhibited by our actuator is comparable to that shown by other actuators, although lower than the theoretical limit of the pneumatic BAM.

**TABLE 1:** Comparison of the actuation parameters of existing encapsulated-hydrogel actuators and the pneumatic BAM against the current work. Where values were not mentioned directly in the text of the referenced work, they have been estimated from available figures.

Actuator	Mode	Contraction/relaxation time	Max. contraction	Max. force
Diteesawat et al. (2021)	Pneumatic	6 s/6 s	∼35%	∼60 N
Santulli et al. (2005)	Hydration (dry-wet)	60 min/NA	>25%	∼15 N
Tondu et al. (2010)	pH	36 min/27 min	13%	80 N
Velders et al. (2017)	Hydration (dry-wet)	7 min/NA	33%	NA
Salahuddin et al. (2020)	Thermal	∼90 min/∼15 min	7.66%	5.14 N
SAP-BAM	Salinity	300 min/45 min	27.5%	10.96 N

The SAP-BAM actuator has been demonstrated in an exclusively aqueous environment (i.e. in transition between salt water and fresh water). In contrast, the actuators presented by Santulli et al. (2005) and Velders et al. (2017) use the faster swelling action seen when a dry gel is first placed into water. Relaxation through dehydration of the gel (which is not demonstrated in those works), is a far slower process. The cyclic speed of actuation of the SAP-BAM is therefore expected to be faster than Santulli et al. (2005) and Velders et al. (2017) but slower than Tondu et al. (2010) and Salahuddin et al. (2020), with the SAP-BAM taking around 5 h to reach 95% of its total contraction in the forward direction, and 45 min during relaxation. However, these times are suitable for use of the actuator in a passive, environmentally-responsive scenario; for instance, this is well within the timeframe required to respond to changes in salinity driven by the tide (with the time between high tide and low tide being approximately 6 hours). In this case the water surrounding the actuator would additionally be flowing, which is likely to decrease the actuation time as salt ions would be removed more rapidly from the immediate vicinity of the actuator by the faster-moving low-salinity water. Likewise, the forces and contractions exhibited by the actuator are appropriate for a passive robot in such a scenario. For the proposed robot with a purpose of periodically releasing some agent into the water, the force need only be sufficient for the opening and closing of some gate or release valve.

The maximum force exhibited by the muscle is on the same order as other encapsulated-hydrogel actuators in the literature. The exception to this is the muscle demonstrated by Tondu et al. (2010), which shows a very high force, but is not passively-driven by the conditions of its environment and instead uses pumps to drive the actuation-inducing pH changes. That work, however, provides a useful indicator for the forces that may be attainable from this type of actuator.

It can be noted that the SAP-BAM actuator can generate forces higher than demonstrated, but that at these forces the actuator begins to leak gel. An issue with the current form of the actuator is therefore the limit of energy that it can absorb before it is irreversibly damaged. As seen in Section 3, this occurs well before bulk failure of the wall material, with small tears in the wall allowing gel to leak from the muscle. As these tears form first around the heat-sealing points, an alternative approach to construction of the nylon-paper composite could mitigate this issue. Alternatively, use of very finely-pored material in place of this composite will allow for a stronger wall, although at the potential cost of a reduced actuation speed. Conversely, a thinner, more porous wall is expected to allow more rapid actuation, but increase the likelihood of the wall splitting under smaller forces.

As well as improving the strength of the actuator walls, future work will investigate alternative geometries using the SAP approach. Many pneumatic muscles other than the McKibben actuator and the BAM can be adapted to be SAP-driven, so long as the internal volume change is finite and the actuator walls can be replaced with a fluid-permeable membrane. For example, the pouch motor presented by Niiyama et al. (2014) is very attractive for such an approach. Indeed, as the swelling of the gel is driven through the walls (as opposed to via an external pressure source), geometries which would be difficult for pneumatic actuators could be used.

Alternatively, a dual-modality actuator using both gel swelling and hydraulics could be used to significantly speed up the response time of the muscle. The body of the actuator could be flooded with pumped fresh or salt water to effect actuation or relaxation respectively (comparable to the pH-varying approach seen in Tondu et al. (2010)). The swelling of the gel could then maintain the actuator in the desired state without requiring the application of constant pressure.

## 5 Conclusion

In this work, we have demonstrated a new form of saltwater-responsive soft artificial muscle driven by the swelling of sodium polyacrylate. Such a muscle responds passively to, and draws actuation energy from, the salt concentration of the water surrounding it, making it appropriate for use in tidal estuaries or similar environments.

The behaviour of this muscle under light load has been characterised, and selected actuation parameters shown to be comparable to other actuators using an encapsulated-hydrogel approach. Future work will focus on strengthening the construction of the artificial muscle, on further modelling the presented actuator in simulation, and on investigation of alternative geometries using a similar approach.

## Data Availability

The datasets presented in this study can be found in online repositories. Data is available at the University of Bristol data repository, data.bris, at https://doi.org/10.5523/bris.2raladk8u6pqu23o1jyc5rueeu.
