# Supplementary material for: Saltwater-responsive bubble artificial muscles using superabsorbent polymers
Source: Front Robot AI. 2022 Aug 29;9:960372. doi: 10.3389/frobt.2022.960372 (PMC9464983; doi:10.3389/frobt.2022.960372)
Supplement: Supplementary file 1 [file DataSheet1.PDF]

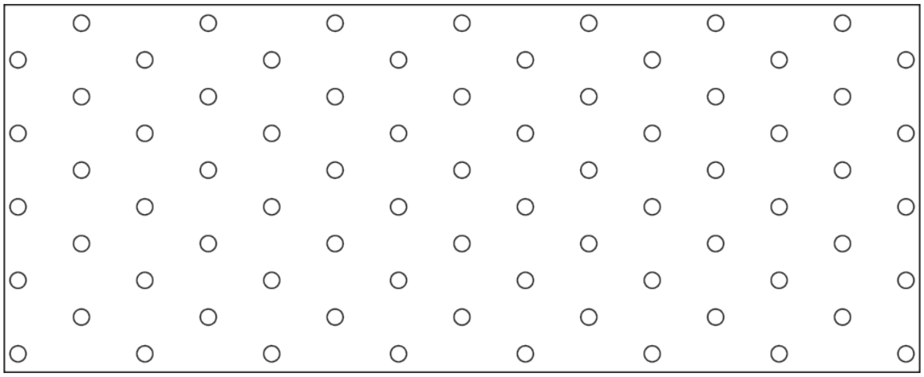

Figure S1: Grid pattern used for heat sealing each nylon-paper sheet used in the construction of a SAP-BAM actuator. The size of each sheet is 80 mm x 200 mm. For sealing, this pattern is repeated in a 2 x 3 grid, and the six samples later separated.
